# Varietal and trait preferences for boiled sweetpotato in urban Hanoi and implications for breeding

**DOI:** 10.1002/fsn3.3774

**Published:** 2023-10-18

**Authors:** Thi Minh Hang Vu, Viet Phu Tu, Diego Naziri

**Affiliations:** ^1^ Hanoi University of Science and Technology Hanoi Vietnam; ^2^ Natural Resources Institute (NRI), University of Greenwich Chatham Maritime UK; ^3^ International Potato Center (CIP) Hanoi Vietnam

**Keywords:** boiled, breeding, CATA, consumer preferences, Hanoi, hedonic test, JAR, sweetpotato, Vietnam

## Abstract

Cognizant of the need to refocus breeding efforts toward end‐product quality traits taking into account the preferences of consumers and in consideration of the rapid urbanization in South‐eastern Asia, this study investigated the consumer's preferences for sweetpotato in Hanoi. Using a mixed‐methods research design, the study identified the most preferred and least preferred attributes of both the fresh and boiled product, disaggregated by gender, age, and socioeconomic status. Preferences and associated traits of six popular varieties were determined. Results indicate that while these varieties largely already meet consumers' preferences, consumers have a clear preference for a few varieties for which marginal adjustments are needed to further increase their acceptability among the growing urban population. Our findings indicate the direction of these adjustments and can contribute to inform demand‐led national and international breeding programs and, ultimately, to higher and faster variety uptake and adoption.

## Graphical abstract

This study investigated consumer's preferences for sweetpotato in Hanoi using a mixed‐methods research design. The most preferred and least preferred attributes of both the fresh and boiled product were identified, disaggregated by gender, age and socioeconomic status. Preferences and associated traits were determined for six popular varieties and recommendations for increased acceptability through breeding programs provided.

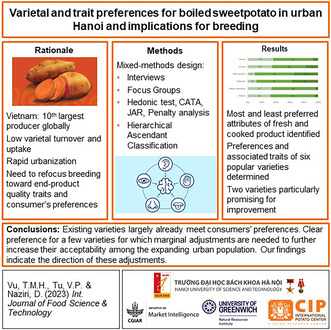

## 1 INTRODUCTION

Over the last decades, major advances in breeding have led to the release of superior productive and nutritious varieties (Herdt & Anderson, 2019; Kholová et al., 2021; Renkow & Byerlee, 2010; Ssemakula et al., 2014). However, breeding programs focused mainly on productivity increase and agronomy‐related characteristics (in particular, resistance to the major biotic and abiotic stresses), while attention to end‐user preferences was not prioritized, resulting in low adoption of improved varieties (Eriksson et al., 2018; Jenkins et al., 2018; Tenkouano et al., 2019).

There is growing recognition of the impelling need to refocus breeding efforts toward end‐product quality traits taking into account the preferences of consumers for quality characteristics (Dufour et al., 2021). Consumer preference for a particular crop variety is often influenced by quality attributes such as appearance, smell, texture, and taste, among others, which must be considered for effective varietal improvement (Siegrist & Hartmann, 2020). Shifting the focus of breeding programs to end‐user preferred traits, especially based on sensory acceptance attributes, has been shown to be a worthwhile strategy to drive demand for new varieties (Baafi et al., 2016; Wismer et al., 2005). In fact, while increased productivity can contribute to improved farmers' livelihoods and incomes, farmers can only achieve these benefits if the varieties they cultivate respond not only to their needs but also possess the traits demanded by consumers and other end‐users in the value chain, such as retailers and processors (Mugisha et al., 2017).

Understanding the trait preferences of these actors is the first step in developing a more demand‐driven breeding program. Furthermore, it is important to investigate gender differences in trait preferences because new varieties can exacerbate gender inequalities and result in negative outcomes for women (Mudege et al., 2021; Tufan et al., 2018). Besides gender, socioeconomic differences such as age and income level, can also influence the preferences for specific traits (Mudege & Grant, 2017; Weltzien et al., 2020).

The relatively poor quality of end products is a particularly common issue across improved varieties of root and tuber crops, translating into low levels of varietal adoption and its subsequent benefits (Mwanga et al., 2017; Thiele et al., 2021).

This study investigates the preferences for boiled sweetpotato of urban consumers in Vietnam, with a specific focus on the capital city, Hanoi. It aims to identify the preferred varieties and the most liked and disliked characteristics of both the fresh and cooked roots in order to inform breeding programs. Attention was paid to the gender and socioeconomic differences of the consumers.

Currently, Vietnam is the 10th largest sweetpotato producer in the world and the third in Asia, after China and Indonesia. In Vietnam, sweetpotato is the most widely cultivated food crop after rice and maize. However, arable land allocated to this crop has drastically declined over the past 20 years, from about 250,000 to 110,000 ha, currently representing about 1% of total cultivated land (FAOSTAT, 2022). Nevertheless, due to its tolerance to heat and drought, and its resilience to extreme weather events (Gatto et al., 2021), the trend is expected to reverse due to climate change, and an expansion of its cultivation is projected in southern and south‐eastern Asia over the next decades (Palao et al., 2019). Despite the reduction in land allocation over the last 20 years, annual harvested output has decreased to a much lower extent, from 1.60 to 1.37 million tons due to a sharp increase in productivity, from 6.3 to 12.5 t/ha (FAOSTAT, 2022). The CGIAR has made an important contribution to the sweetpotato breeding program in Vietnam and the introduction of higher yielding varieties. According to Gatto et al. (2018), Vietnam has released 19 improved varieties, five of which being related to the International Potato Center (i.e., selected from CIP crosses or crossed from CIP progenitors) and about 20% of the total sweetpotato land is presently planted with CIP‐related varieties.

The average per capita annual consumption of sweetpotato in the country is estimated at 4.7 kg. The study selected boiled products because the most common mode of consuming sweetpotato in Vietnam is in boiled form.

This study aims to address the following research questions:

How do Hanoians consume sweetpotato and what are their most preferred and least preferred characteristics of the fresh and boiled product, disaggregated by gender, age, and socioeconomic status?

What are the consumers' preferences for each variety and their associated characteristics?

How can the most popular sweetpotato varieties in Vietnam be improved through breeding to better respond to the preferences of the expanding urban population and, in turn, increase variety uptake and adoption?

To our knowledge, this is the first quantitative study assessing consumer's varietal and trait preferences for sweetpotato in Vietnam. Information obtained underpins end‐user‐oriented and socially inclusive breeding strategies. The study findings are expected to inform the integration of consumer's preferences and needs in sweetpotato breeding programs, thus guiding the selection criteria for breeding. As proposed by Dufour et al. (2021), the identified quality characteristics of boiled sweetpotato can be associated with biochemical and physical traits of fresh materials, which can be used to refine Target Product Profiles (Donovan et al., 2022) and shared with breeders to develop improved selection tools. Physicochemical scientists, food technologists, and breeders then can jointly develop and implement high‐throughput phenotyping techniques (e.g., near‐infrared spectroscopy—NIRS) to screen large numbers of potentially improved genotypes for developing superior varieties for both producers and end‐users. New varieties that meet the demand of consumers will be more likely to be adopted at scale and benefit all stakeholders in the value chain.

## 2 MATERIALS AND METHODS

The study used a mixed‐methods research design by adapting the methodology developed by Forsythe et al. (2020) to the specificities of urban consumers. The design included Focus Group Discussions (FGDs) and consumer tests. Before consumer tests, structured individual interviews were conducted to collect data on the socioeconomic characteristics of respondents.

### 2.1 Focus group discussions

#### 2.1.1 Group composition

Sixty‐four people were purposively selected to ensure diversity in gender, age, and income level. Based on this, participants were distributed into eight groups (Table 1).

**TABLE 1:** Grouping of FGD participants.

Group	Gender	Age	Age group	Household income (VND/month)	Income group
1.	Male	<25	Young	>20,000,000	High
2.	Male	<25	Young	≤20,000,000	Low/Medium
3.	Male	25–45	Middle‐aged	>20,000,000	High
4.	Male	25–45	Middle‐aged	≤20,000,000	Low/Medium
5.	Female	<25	Young	>20,000,000	High
6.	Female	<25	Young	≤20,000,000	Low/Medium
7.	Female	25–45	Middle‐aged	>20,000,000	High
8.	Female	25–45	Middle‐aged	≤20,000,000	Low/Medium

*Note*: 1 USD = 23,691 VND (as of 9/2/2023).

#### 2.1.2 Procedure

The FGDs were carried out according to the guidelines developed by Simon (1999). Each FGD was conducted in participants' native language and lasted 40–60 min. The discussion was facilitated by a trained moderator who followed established guidelines and ensured the active contribution of all participants. During the process, boiled sweetpotato samples were served for testing. The discussion was recorded, and notes were taken.

#### 2.1.3 Data analysis

The voice recordings were transcribed, and the themes elicited by the moderator and the note‐taker. The descriptors mentioned in the FGDs were extracted and used for the CATA questions (see Section 2.2.3).

### 2.2 Consumer test

#### 2.2.1 Material

The six most common sweetpotato varieties found in markets and farmer fields in and around Hanoi were collected (Table 2). Roots of each variety were boiled, cut in 50 g slices, packed in a covered plastic box to retain the odor, and stored at room temperature. The samples were served in paper plates and randomly assigned three‐digit codes. All experiments were carried out in equipped standardized booths at the Sensory Laboratory of the Hanoi University of Science and Technology (HUST).

**TABLE 2:** Sampled sweetpotato varieties.

No.	Name of variety	Location of collection	Origin	Price (VND/kg)
1	Red Hoang Long	Farmer field in Ba Vi district	Ba Vi—Vietnam	12,000
2	White Hoang Long	Roadside retailer in Ba Vi district	Ba Vi—Vietnam	14,000
3	Beniazuma (Japanese variety)	Roadside retailer on AH13 road (Hoa Binh)	Son La—Vietnam	16,000
4	Khoai Mat	Market retailer in Hanoi	Dak Lak—Vietnam	25,000
5	Khoai Bo	Market retailer in Hanoi	Dak Lak—Vietnam	25,000
6	Unknown (imported from China)	Market retailer in Hanoi	China	40,000

#### 2.2.2 Panel

Eighty participants (45 women and 35 men) were recruited from the HUST network and database. Composition by gender and age is presented in Table 3. Half of participants indicated a monthly household income lower than VND 3 million, 38% between 3 and 15 million, and 12% above 15 million. Fifty‐five percent were single, 39% married with children, and 5% married without children.

**TABLE 3:** Gender and age of consumer test participants.

	18–23 years old	24–30 years old	31–40 years old	>40 years old	Total
Men	8	7	11	9	35
Women	13	9	11	12	45
Total	21	16	22	21	80

#### 2.2.3 Procedure

Each participant was first interviewed using a semi‐structured questionnaire and then subjected to the following standard methods for consumer test.

##### Hedonic test

The hedonic test was conducted according to the ISO 4121:^2003^ and ISO 11136:^2014^ guidelines. Samples were coded and presented one by one according to the Williams Latin Square method. The panelists received no information on the tested products before and during the test. In line with similar studies (cf. Granato et al., 2010; Lim et al., 2022), panelists were asked to score their general liking on a 7‐point scale (from 1 = “I dislike very much” to 7 = “I like very much,”, 4 = “Neither like nor dislike”). Samples with average liking score higher than 4 were considered to be accepted by consumers. In addition to general liking, participants were also asked about their preference in terms of specific attributes, i.e., odor, taste, texture and after taste. Mineral water was used to rinse the mouth between consecutive samples.

##### Check‐All‐That‐Apply test (CATA)

The CATA test was carried out in accordance with standard methods (Ares, 2015). Terms extracted from FGDs were used in the test, and consumers were asked to check the terms they found most appropriate to describe the samples. This allows to elicit the respondent's perception of each sample and explain its acceptability level.

##### Just‐About‐Right test (JAR)

Following the methodology proposed by Lawless and Heymann (1998), consumers were invited to test the products and rate the intensity of each attribute on a 3‐point JAR scale with a central score of ‘just about right’ (too little/just about right/too much). This allows to determine the attributes that needed to be adjusted as well as the direction of adjustment (increase or decrease) to maximize consumers' satisfaction.

#### 2.2.4 Data analysis

##### Hedonic test

The hedonic scores were subjected to a two‐way ANOVA with the following model, with “panelists” as random factor.

Score=panellists+product+productxpanellists+error

When significant effects occurred, a mean comparison LSD test was performed. The results were also processed by Hierarchical Ascendant Classification (HAC) algorithm to classify participants according to their liking scores and identify clusters with similar preferences.

##### CATA test

Correspondence analysis was conducted to generate a frequency matrix of CATA descriptors. This analysis allowed to visualize the set of data in two‐dimensional graphical form and define the sweetpotato products space by identifying the relationship between the terms from the *CATA and the samples* (Beh & Lombardo, 2014; McEwan & Thomson, 1989).

##### JAR test

Penalty analysis was carried out to identify the factors that contribute to the greatest reduction in product satisfaction, i.e., the possible penalty paid by the product in terms of reduced overall liking for not being “just about right” on a characteristic (Lawless & Heymann, 1998). The penalty is often referred to as “mean drop on overall liking.” This analysis allowed to identify the attributes that need improvement and suggest directions for the improvement of each sweetpotato product.

## 3 RESULTS

### 3.1 Focus group discussions

#### 3.1.1 Uses and perception of sweetpotato

About 31% of respondents indicated that on average they consume sweetpotato once per month, 36% two to three times a month, 26% one to two times a week, and 7% three or more times a week. Sweetpotato is eaten mainly as snack or side dish. Boiled and steamed roots are the most common forms of consumption (92% of respondents), and they are usually prepared at home. Other common ways to consume sweetpotato, particularly by young people, include grilled, fried, and fritters which are often purchased by street vendors. Less frequently, sweetpotato is consumed in soups, curries, dried, or even raw.

Respondents perceive sweetpotato as a tasty, fulfilling, and healthy food that is easy to prepare. On the other hand, they reported some issues (perishability and limited appeal to some children) and beliefs (toxicity of sprouted roots) that could limit its consumption (Table 4). The reported limited appeal on children was not expected since other studies have not found differences in sweetpotato acceptability between mothers and their preschool children (Tomlins et al., 2007).

**TABLE 4:** Consumer's general perception about sweetpotato.

Social representation	Very good for health: help lose body weight (especially if boiled), good for digestion Feel comfortable and light after consuming it Tasty and easy to make full
Use and attitude	Not an everyday food, eaten when available and for pleasure A snack food or side dish Can be eaten with other foods Eaten in the morning or at noon to skip breakfast and/or lunch Can substitute rice if on a diet Easily available in the market Easy to boil, grill, and even to cook in microwave oven Not easy to keep it for a long time Toxic when sprouting Some children do not like it
Product and market experience	Popular, cheap Not cheap in off‐season, 1 kg of roots equal to 2 kg of rice Japanese variety expensive, often >20,000 VND/kg Potato is cheaper and easier to eat than sweetpotato Rich variety in sensory quality: from mealy to puree, from sweet (as honey) to tasteless, from yellow to pale

Consumers also appear to be sensitive to market prices, which can be higher than rice and potato in the off‐season. About 54% of respondents indicated price as the main reason for consuming sweetpotato as a substitute for other foods and indicated an acceptable price in the range of 15,000–20,000 VND/kg.

#### 3.1.2 Most and least preferred varieties

FGD participants indicated the most and least preferred sweetpotato varieties for different preparation methods. Most participants, particularly men, were unable to name the varieties and referred to them by the color of the flesh and/or peel. For boiling, both men and women prefer a wide range of varieties of different colors. Strong taste, sweetness, and mealiness are key attributes of the most liked varieties (Table 5). Mealiness is a common sought‐after characteristic in several countries in the Global South (cf. Mwanga et al., 2021), while preference for sweet varieties can differ considerably, with consumers in some countries preferring non‐sweet varieties (Baafi et al., 2016). Poor taste is the main characteristic of the least preferred varieties (Table 6). There is strong agreement between women and men that the variety Khoai Mat is suitable for grilling but not for boiling.

**TABLE 5:** Preferred varieties by gender.

Preparation	Men	Reason	Women	Reason
Boiled	Purple sweetpotato (flesh and peel) Orange sweetpotato Sweetpotato with purple peel and yellow flesh White sweetpotato	Delicious, sweet, mealy	Sweetpotato from Da Lat (yellow or purple flesh) Japanese sweetpotato (yellow) Sweetpotato with purple peel and white flesh (taste like Japanese)	Delicious snack Mealy, sweet, slightly dry
Grilled	Khoai Mat (sweet, soft, and puree)	Soft	Khoai Mat (sweet, soft, and puree)	

**TABLE 6:** Least preferred varieties by gender.

Preparation	Men	Reason	Women	Reason
Boiled	Khoai Mat	Puree	Purple sweetpotato	Taste not good if with umami taste
			Khoai Mat	Puree, too soft

#### 3.1.3 Most and least preferred characteristic of fresh roots

**FIGURE 1:**
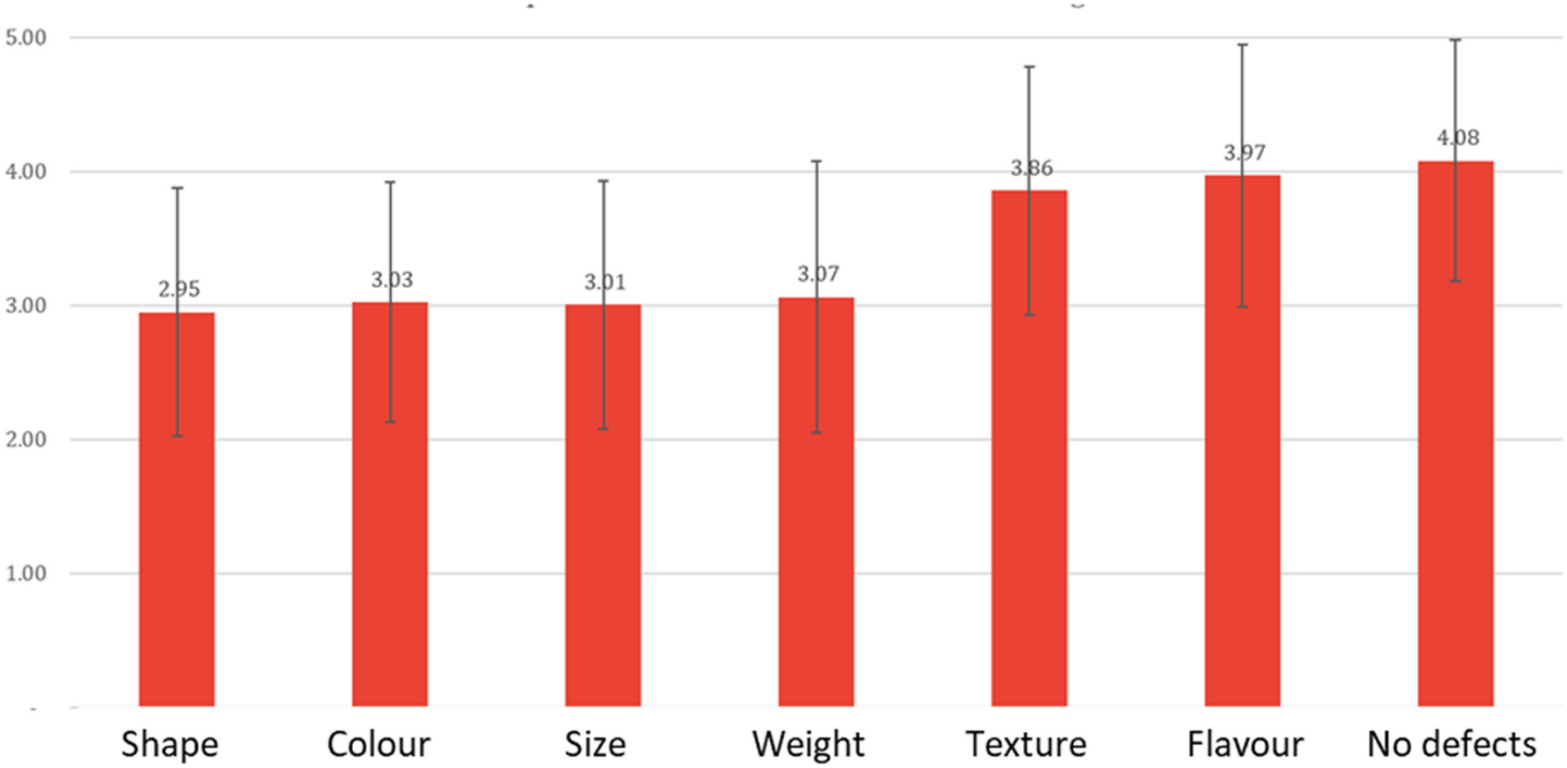
Main criteria for selecting sweetpotato roots.

The vast majority (82%) of the respondents indicated that the main criteria for selecting fresh sweetpotato in the market is root quality. Similar to what other studies have found, preferences for fresh roots are primarily associated with morphological and physicochemical characteristics (Mwanga et al., 2021). Lack of external defects (holes, black spots, cuts, and bruises) is the main sought‐after characteristic, followed by flavor, texture, weight, size, color, and shape of the root (Figure 1). However, the relative importance of these traits is different from what found in other countries. For example, Mwanga et al. (2021) observe that in Uganda, large and hard roots are by far the most important attributes when selecting fresh sweetpotato for boiling.

Table 7 shows the gender differentiated preferred characteristics when purchasing roots. Both women and men prefer medium to big size roots (with men preferring larger ones), with elongated shape, smooth peel, and lack of defects. Women seem to pay more attention to peel color (purple or yellow, which can be associated with specific varieties) and were able to clearly state the reason for their preferences. Men are more attracted to the firmness of the root. Similarly, women and men reported comparable least preferred characteristics for fresh roots, with women more attentive to peel color (dislike white) and men to the softness of the root (Table 8).

**TABLE 7:** Gender‐differentiated preferred characteristics when buying fresh roots.

	Men		Women	
	Characteristics	Reason	Characteristics	Reason
Size	Big, but not too big Middle part is moderately big	The size should fit the mouth when biting Big roots are more suitable for frying	Medium, not too big	
Shape	Elongated or round shape		Elongated	
Surface	Smooth peel No black spots Looks fresh Clear color Firm at touch	Soft indicates that it has been stored for a long time, spoiled	Smooth peel Purple or yellow color Uniform color No black spots	Too light color linked to bland taste Too dark color linked to bed smell (like alcohol) when boiled

**TABLE 8:** Gender‐differentiated least preferred characteristics when buying fresh roots.

	Men		Women	
	Characteristics	Reason	Characteristics	Reason
Size	Too big	Long time to boil Unevenly cooked	Too big Too short	Hard to boil/cook
Shape	Folded shape		Humpback shape	Difficult to eat, difficult to steam, fibrous
Surface	Soft, rotten Bruised or rough peel Black spots Signs of bugs and weevils (holes) Cuts and bruises	Stale After boiling, the roots are hard and not good to eat	Roots look old White Strange color Holes, signs of pests, weevil	Too fibrous roots with white peel will be bland Roots damaged by weevils and other pests will be bitter

Although no major differences were observed between different income and age groups, women could share much more specific and detailed criteria for choosing sweetpotato in the market whereas most men seem to be just “eaters.” Two women said that they preferred roots of the same size: “If sweetpotatoes have different sizes, when I boil them, I have to take out the small roots first and then continue to boil the large ones for a longer time.” The ability of women to indicate a higher number of characteristics important for ensuring a good, high‐quality product relate to their expertise in preparing boiled sweetpotato, as also suggested by Mwanga et al. (2021). In general, men rarely buy sweetpotato in the market (on average, 2–3 times/year, 1–2 roots/time), rely more on the seller's choice, and seem less interested in the price. Higher income women pay more attention to defects (withered, wrinkled, and dark peel; presence of shoots, holes, and soil), while lower income ones are more demanding in terms of peel color and firmness of the root.

#### 3.1.4 Most and least preferred characteristic of boiled sweetpotato

Respondents described the most liked characteristics for a boiled sweetpotato root. Both women and men indicated mealiness and sweetness among the three most important characteristics. These findings are largely consistent with what found by many scholars in sub‐Saharan countries: mealiness, sweet taste, and good smell are important characteristics for boiled sweetpotato (Mwanga et al., 2016, 2021; Tomlins et al., 2012; Tumwegamire et al., 2011). However, as previously reported, sweet taste is not a preferred trait in all countries (Baafi et al., 2016). In addition, we found that women prioritized its smell and flavor, while men its softness (Table 9). Unsurprisingly, women were able to indicate more descriptors and way to recognize sought‐after characteristics. Specifically, women were also interested in the color of the flesh and the size of the root. Men were able to assess the quality of the food only after tasting it, while women were able to detect it also by smelling and handling of the root.

**TABLE 9:** Gender‐differentiated preferred characteristics of boiled sweetpotato.

Description of high‐quality boiled sweetpotato	Three most important characteristics	How to recognize those characteristics
Men		
Mealy Sweet Color uniformity Soft Hot	Sweet Mealy Soft	Need to taste
Women		
Good flavor Flesh with yellow bean color No fiber Sweet Mealy Light colored flesh Big roots Burnt roots at bottom of the pot	Mealy Sweet Good smell and flavor	Easy to break Taste Smell

More marked differences between women and men were reported regarding the least liked characteristics (Table 10). Women indicated that they dislike roots that are bruised inside, fibrous, wet, too mushy, or with an unpleasant taste or smell. Men particularly dislike roots that are not properly cooked, fibrous, too powdery, not mealy, and with a bland taste. While some of these characteristics can be associated (e.g., too mushy and overcooked), it is interesting to note the different ways in which they are described. Again, women indicate a broader set of senses used to detect the least preferred characteristics.

**TABLE 10:** Gender‐differentiated least preferred characteristics of boiled roots.

Description of low‐quality boiled sweetpotato root	How to recognize those characteristics
Men	
Overboiled, cracked, not well cooked Fibrous Bland, not sweet Not mealy Weevil damage Too powdery	Need to taste
Women	
Dark bruises inside Fibrous Wet on the surface Too mushy (boiled for too long) Bitter Smell of alcohol Weevil damage	Break and observe Taste Smell

### 3.2 Consumer test

#### 3.2.1 Hedonic test

Statistically significant differences (*p* < .001) were found among the average liking scores of the sweetpotato varieties' for four aspects: General liking, Appearance, Flavor and smell, and Texture.

**FIGURE 2:**
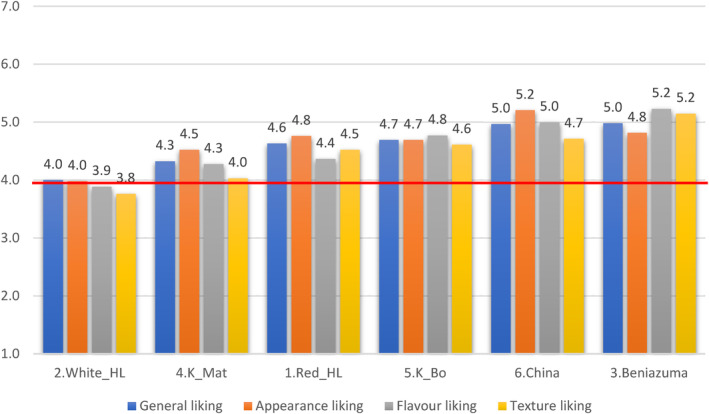
Average liking scores of sample varieties.

Variety Beniazuma had the highest General liking score, followed by sweetpotato imported from China and Khoai Bo (Figure 2). However, Beniazuma and sweetpotato from China showed no significant difference in mean score. White Hoang Long appears as the least liked variety.

**FIGURE 3:**
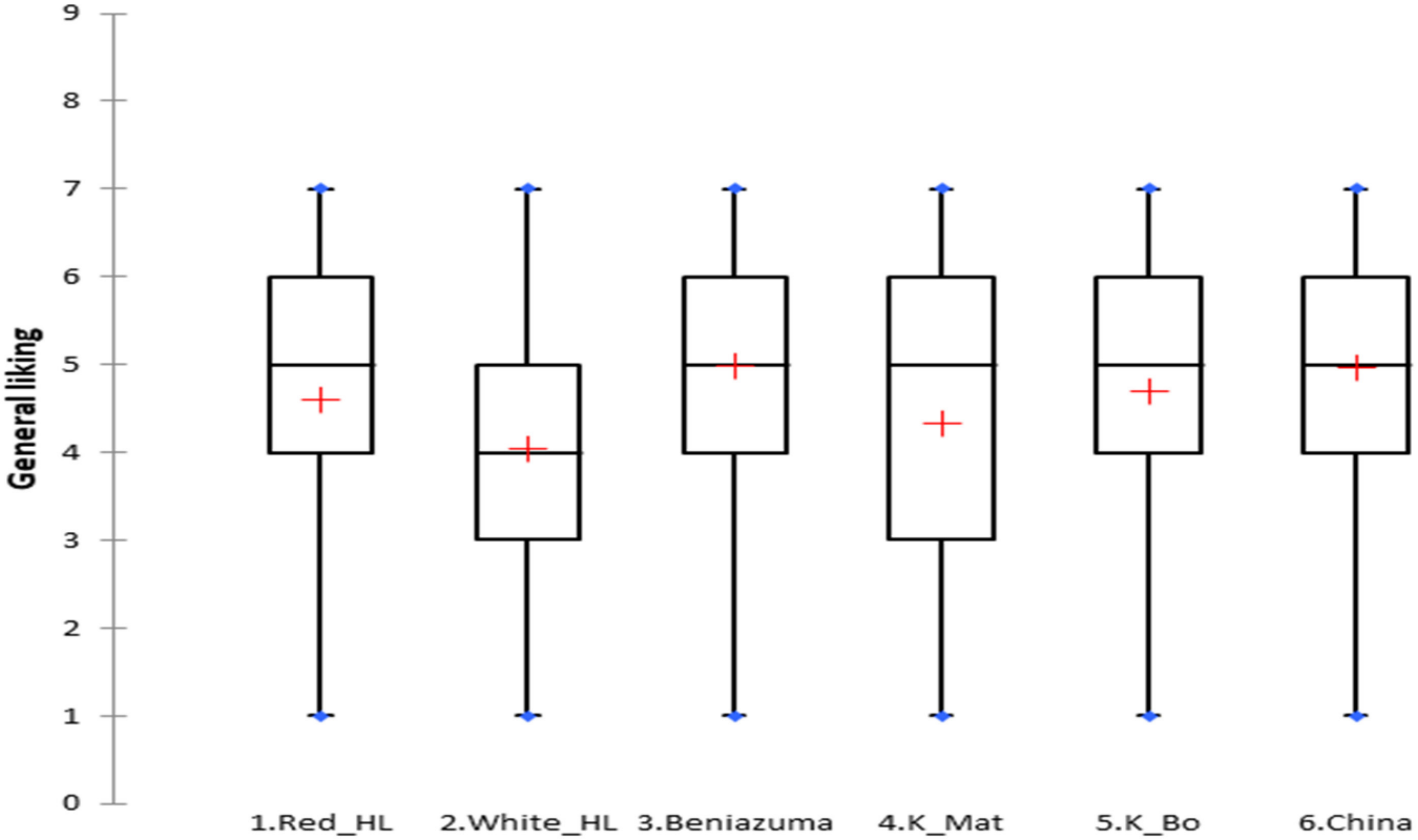
Boxplot chart of general liking.

As shown by the boxplot chart (Figure 3), approximately 75% of the respondents rated Red Hoang Long, Beniazuma, Khoai Bo, and sweetpotato from China in their liking region (>4 points). About 50% of the respondents rated White Hoang Long below the acceptable range (<4 points). Khoai Mat shows high heterogeneity, with 50% of respondents indicating high appreciation for it (≥5 points) and 25% disliking it (≤3 points). This resulted in an average liking score well below the median.

**FIGURE 4:**
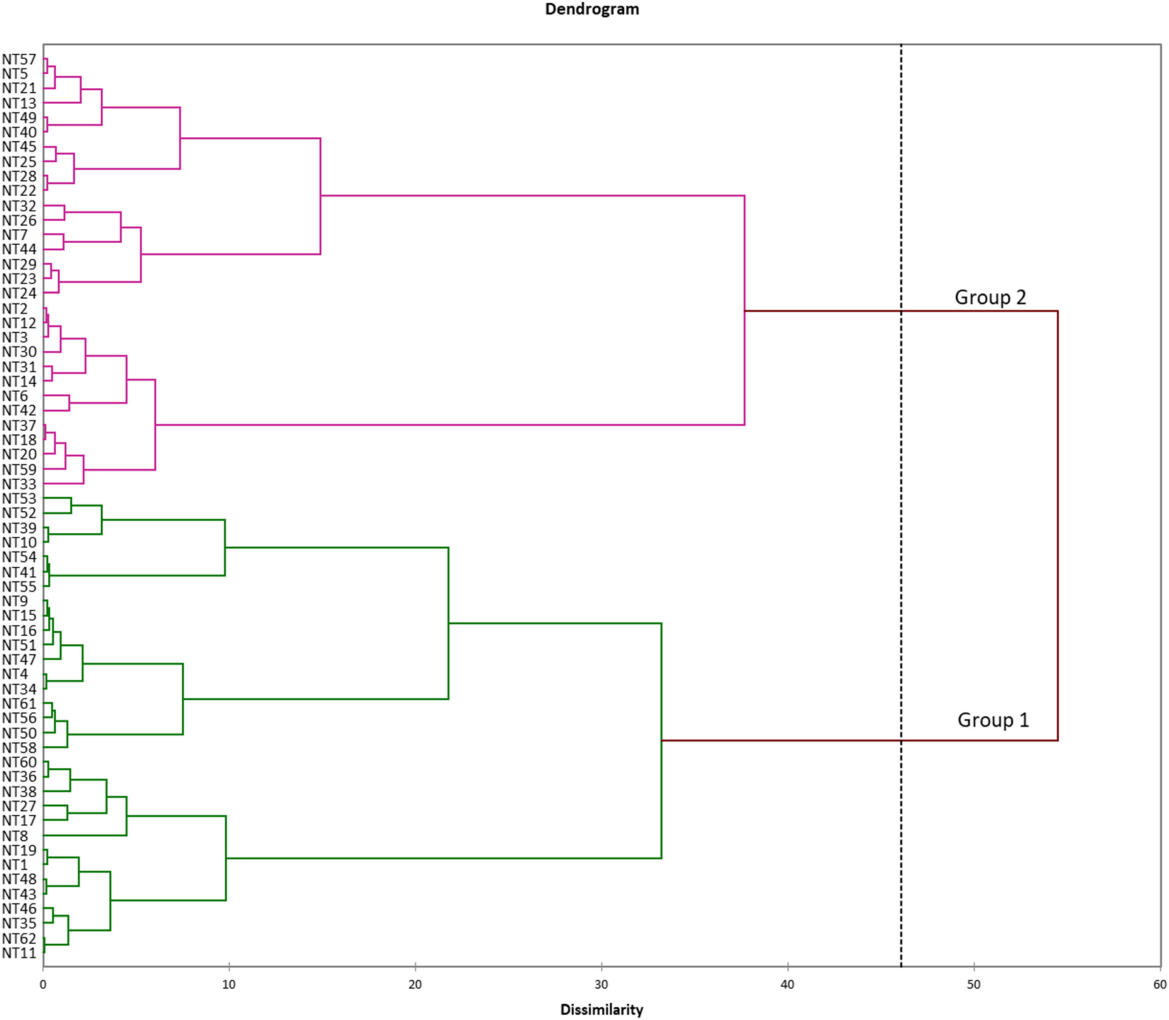
HAC results of consumer segmentation.

Hierarchical Ascendant Classification (HAC) allowed to cluster participants into two groups, with individuals in each group having similar preferences for the sweetpotato varieties (Figure 4). Table 11 shows the average hedonic scores for each of the target varieties. Major differences between group 1 and group 2 were found, with group 1 preferring sweetpotato from China and group 2 preferring Beniazuma.

**TABLE 11:** Average hedonic score (on a 7‐point scale) by cluster.

Group	Red_HL	White_HL	Beniazuma	K_Mat	K_Bo	China
1	4.786	4.385	4.374	3.971	4.270	5.609
2	4.395	3.861	5.633	4.326	4.866	4.267

As shown in Table 12, group 1 consists primarily of single young people with low income, and group 2 of older consumers with higher income and married. No major differences in gender representation were found between the two groups.

**TABLE 12:** Characteristics of clusters.

	Group 1 (%)	Group 2 (%)
Age		
18–23	**57**	33
24–30	3	8
>30	40	**59**
Gender		
Male	49	40
Female	51	60
Income (VND/month)		
<3 m	**57**	23
3–4.5 m	6	5
4.5–7.5 m	6	3
7.5–15 m	28	25
>15 m	3	**46**
Marital status		
Single	**63**	45
Married with children	28	**53**
Married with no children	6	2
Other	3	0

*Note*: In bold, the main sociodemographic characteristics of the cluster.

#### 3.2.2 CATA

**FIGURE 5:**
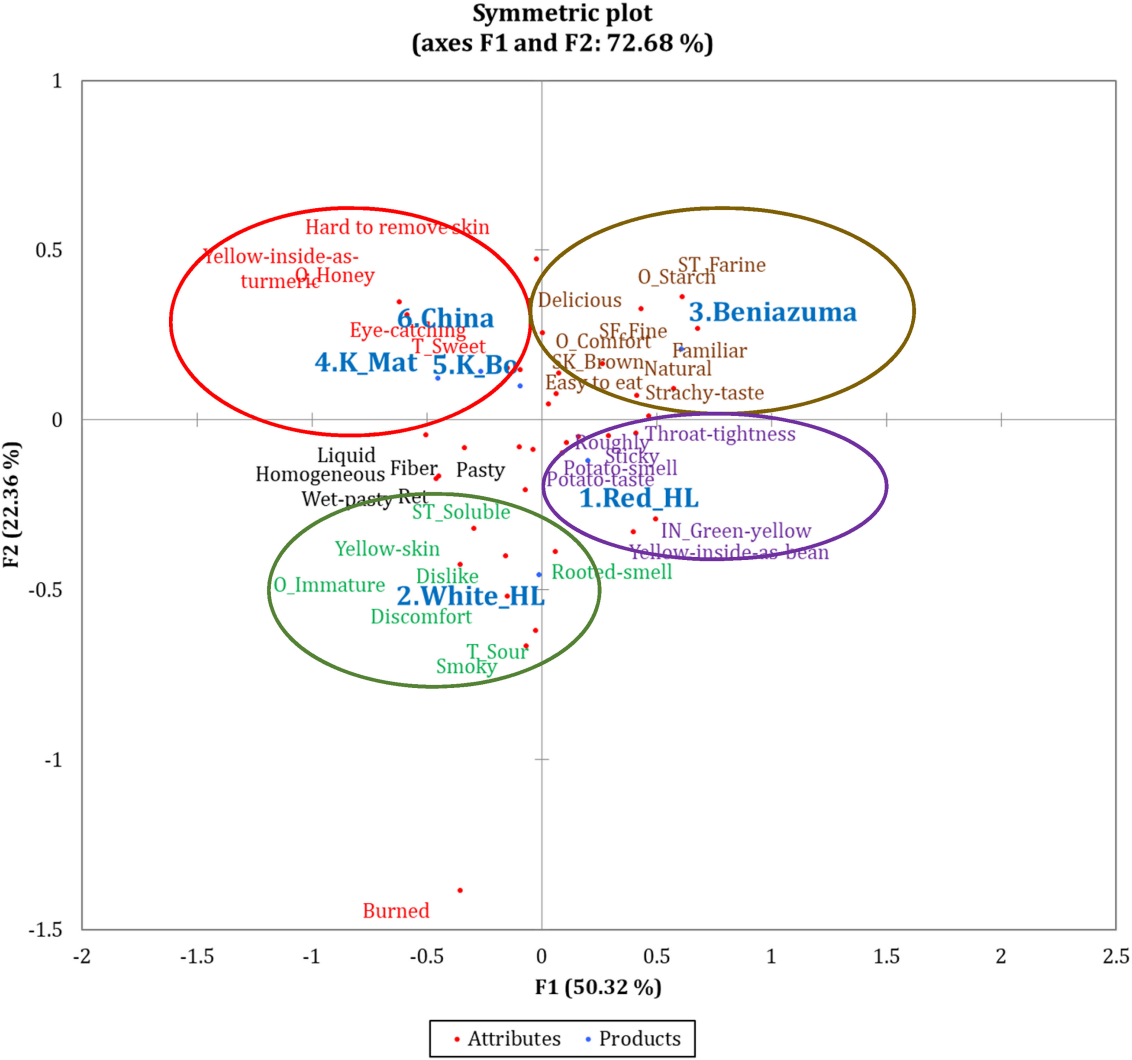
Sweetpotato products space and properties described by panel.

Figure 5 shows the CATA map, which graphically represents the characteristics of each variety based on the frequencies of citation of sensory and perception descriptors. The closer the varieties, the more similar they are. The results of the correspondence analysis on the CATA dataset allowed to identify four groups of varieties with similar sensory properties:

- Group 1 *(highlighted in green)*: White Hoang Long—(characterized by) *Dissolving, Yellow peel, Dislike, Immature smell, Discomfort, Sour, Smoky, Rotten smell*
- Group 2 *(highlighted in purple)*: Red Hoang Long—*Throat tightness, Roughly, Sweetpotato smell, Sweetpotato taste, Yellow‐inside‐as‐bean*.
- Group 3 *(highlighted in brown)*: Beniazuma—*Crumbly, Starchy smell, Fine surface, Comfort‐smell,* 1 *Familiar, Natural, Starchy taste, Brown peel, Easy to eat, Delicious*.
- Group 4 *(highlighted in red)*: China, Khoai Bo, Khoai Mat—*Hard to remove peel, Honey smell, Yellow‐inside‐as‐turmeric, Eye catching*.

These findings can be used to interpret the results of the hedonic test and to preliminary identify the key sensory attributes that made a certain variety the most (or least) preferred by the panelists. As presented in the previous section, Beniazuma and sweetpotato from China were the most preferred varieties. In the case of Beniazuma, this is likely associated with its crumbly texture, comforting starchy smell, and delicious starchy taste. Sweetpotato from China is probably preferred due to its eye‐catching appearance and honey‐like smell. Conversely, White Hoang Long was the least preferred variety, probably due to its sourness and its immature, smoky, and rotten smell.

#### 3.2.3 JAR

**FIGURE 6:**
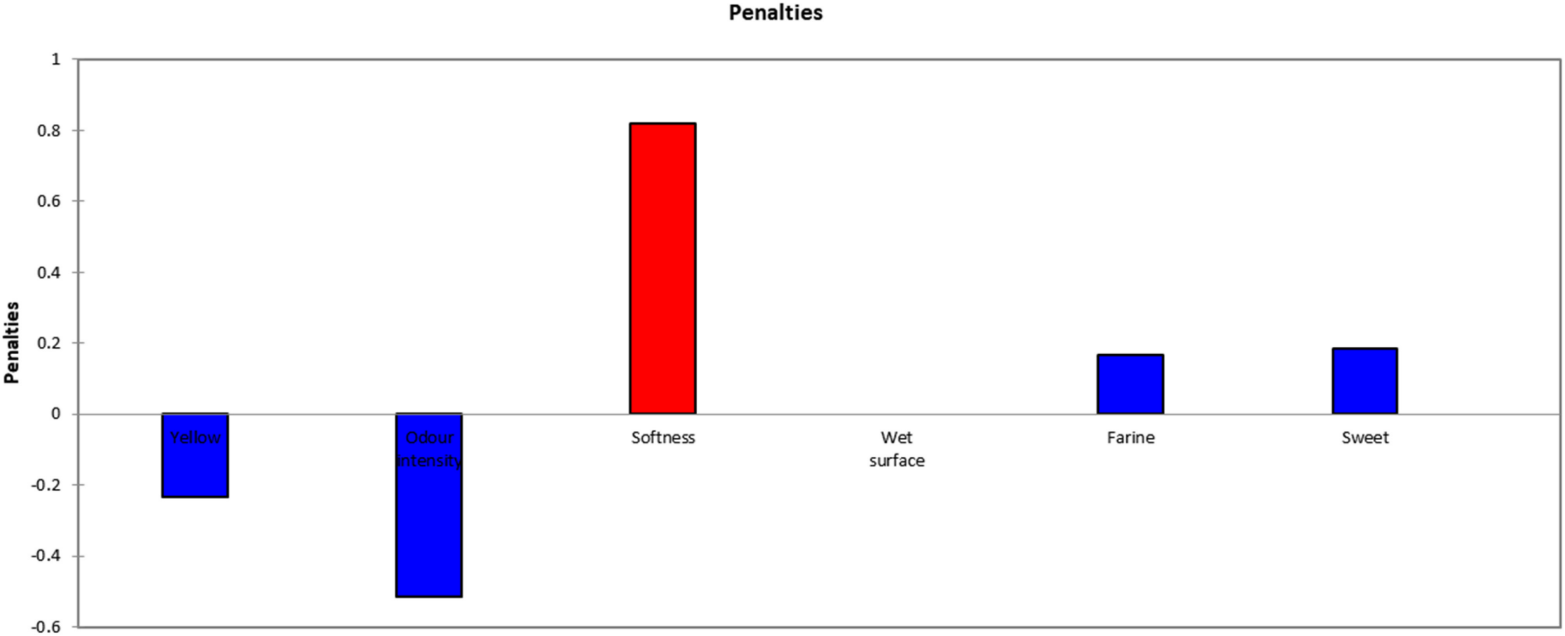
Penalty chart of Beniazuma.

The JAR analysis allows to orient the improvement by identifying how key attributes need to be adjusted to meet consumer's preferences. Six attributes were considered: Yellow color, Color intensity, Odor intensity, Softness, Wet surface (sap), Mealiness, and Sweetness. The detailed JAR results for each variety are presented in the Appendix 1. For each variety, a penalty analysis was conducted to analyze the relationship between the intensity score of the attributes on the JAR scale and the General liking score for the variety. The difference in the JAR score was only significant if it was linked to a statistically significant reduction in the General liking score. Using a critical level of 20% of respondents and the *p*‐value of penalty ≤.05, significant attributes that need adjustment were identified. The penalty chart for the variety Beniazuma (Figure 6) shows that its softness was found to be too low by a significant proportion of panelists and reduced the General liking score by 0.897 points. The variety was also found too sweet; however, this does not significantly affect the hedonic score. The penalty charts of other sampled varieties are presented in the Appendix 2. Table 13 presents a summary of the main results and shows the attributes that reduced the General liking score of a variety in a statistically significant way and, therefore, to be adjusted in order to improve the consumer's acceptability.

**TABLE 13:** The *p*‐value of penalty analysis.

	Beniazuma	China	K_Bo	Red_HL	White_HL	K_Mat
Yellow color	.543	.378	.606	.008	**.033**	**.025**
Odor intensity	.190	.239	.230	**.036**	.297	**.042**
Softness	**.036**	.456	**.019**	.746	.557	.888
Sap	–	**.028**	.156	**.027**	.210	.329
Mealiness	.666	.067	.454	.118	.427	.435
Sweetness	.642	.106	.326	.095	.283	.597

*Note*: In bold, significant JAR attributes in need of adjustment, *p*‐value <.05.

For the sweetpotato imported from China, the sap seeping out of the sliced roots (wet surface) was deemed too much, and this affected the preference of consumers for this variety. The variety Khoai Bo was found too soft, while in the variety Red Hoang Long the odor intensity and the sap were too little and significantly affected the liking score. The flesh color of variety White Hoang Long was not yellow enough to meet the preferences of consumers. Conversely, the variety Khoai Mat was assessed as being too yellow, which may be considered as odd and unnatural. Furthermore, its odor intensity was found excessive.

## 4 CONCLUSIONS

Cognizant of the need to refocus breeding efforts toward end‐product quality traits taking into account the preferences of consumers for quality characteristics (Costell et al., 2010; Dufour et al., 2021), this study investigated the preferences for boiled sweetpotato of urban consumers in Vietnam, with a focus on the capital city Hanoi. Sweetpotato is perceived as a tasty, fulfilling, and healthy food that is easy to prepare. However, its perishability, limited (and unexpected) appeal on children, and some (unfounded) beliefs about the toxicity of sprouted roots are considered negative attributes.

The quality of the root is the main criteria for selecting fresh sweetpotato in the market. Lack of external defects is the main sought‐after characteristic, followed by flavor, texture, weight, size, color, and shape of the root. Medium to big size roots, with elongated shape, smooth peel, and lack of defects are the most preferred by both women and men. Some gender differences emerged, with women, usually in charge of the purchase, preferring purple or yellow skin color and men very firm roots.

With regard to the boiled product, mealiness and sweetness are among the top three most important characteristics for both women and men. Besides, women prioritize smell and flavor, while men softness.

The preferred characteristics for both fresh roots and boiled sweetpotato are largely similar to those reported by the few related studies conducted in other countries in the Global South. However, some important differences emerged. In sub‐Saharan countries, consumers prioritize the size and firmness of the root (which is perceived as linked to freshness and longer shelf‐life), while quality‐related attributes are more sought after in Hanoi. This might be related to difference in socioeconomic status and food security. With regard to the boiled product, mealiness is consistently reported as a preferred characteristic in literature, while sweetness and softness are actually nonpreferred traits in several countries. These differences confirm that consumer preferences are context‐specific and findings in one country are likely not to be applicable in other geographies.

Overall, women were able to indicate more descriptors and a broader set of senses used to detect the most and least preferred characteristics for both fresh and cooked roots. This likely relates to their prominent role in choosing, purchasing, and cooking sweetpotato. These findings confirm the importance of using a gender lens when conducting consumer preference studies as female and male consumers might have different roles in purchasing, preparing, and consuming the food and, hence, different preferences.

Second, we determined the consumers' preferences and associated characteristics of the six most common sweetpotato varieties found at the time of the study. Although all varieties were in the liking region of the hedonic scale, two varieties stood out: Beniazuma and sweetpotato imported from China. Beniazuma is the preferred choice for older consumers with higher income and married, while the Chinese variety is preferred by single youth with low income. Beniazuma is most likely preferred because of its starchy texture, smell and taste, while sweetpotato from China because of its eye‐catching appearance and honey‐like smell. However, a quantitative descriptive analysis (QDA®) should be carried out in future studies to gain a deeper understanding of the relationship between sensory properties and hedonic preference of each variety.

Third, we identified how specific attributes in existing varieties could be adjusted to better respond to the preferences of Hanoian consumers. With regard to the two most preferred varieties, only one characteristic needs to be adjusted for increasing their acceptability: the texture of Beniazuma should be softer while the sap of the Chinese variety should be reduced. In the case of Beniazuma, the result is not surprising as this variety has been developed for Japanese consumers for whom dry mouth‐feeling is a key trait (Tan et al., 2007). Two more varieties would require changing one single attribute only, more yellow flesh (variety White Hoang Long) or firmer texture (variety Khoai Bo). However, considering the high investment and long time required to improve varieties through breeding, adjusting these two varieties might not be the best option, since the former is the least preferred variety overall, and the latter is the second to last preferred variety by the younger generation. The other two varieties examined would require the adjustment of at least two attributes which, due to the high complexity of breeding for vegetatively propagated crops (Bonierbale et al., 2003), might be challenging in face of scarce resources and longer time required.

In conclusions, although popular sweetpotato varieties marketed in Hanoi largely already meet consumers' preferences, consumers show preference for a few varieties for which marginal adjustments are needed to increase their acceptability among the expanding urban population. Our findings indicate the direction of these adjustments and can contribute to inform demand‐driven national and international breeding programs. Specifically, they can help screening sweetpotato populations at earlier stage of the breeding pipeline, including through refinement of target product profiles and enhanced calibration of improved selection tools such as high‐throughput phenotyping techniques. It is expected that this will contribute to higher and faster variety uptake and adoption and ultimately increase food security and livelihood opportunities in the country.

## AUTHOR CONTRIBUTIONS

**Thi Minh Hang Vu:** Conceptualization (equal); data curation (equal); formal analysis (equal); investigation (equal); methodology (equal); writing – original draft (equal); writing – review and editing (equal). **Viet Phu Tu:** Conceptualization (equal); data curation (equal); formal analysis (equal); investigation (equal); methodology (equal). **Diego Naziri:** Conceptualization (equal); funding acquisition (lead); investigation (equal); methodology (equal); project administration (lead); writing – original draft (equal); writing – review and editing (equal).

## FUNDING INFORMATION

This work was supported by the CGIAR Research Program on Roots, Tubers and Bananas (RTB) and the One CGIAR Market Intelligence initiative.

## CONFLICT OF INTEREST STATEMENT

The authors declare that they have no known competing financial interests or personal relationships that could have appeared to influence the work reported in this paper.

## ETHICS STATEMENT

The experiments were carried out in the sensory laboratory of the Hanoi University of Science and Technology (HUST) and conducted in accordance with the University's ethical guidelines for scientific research. The participants were informed about the testing procedure and asked to give their written informed consent prior to participating in the study.

## Data Availability

Data supporting the findings of this study are available from the corresponding author on a reasonable request.

## Footnotes

^1^
Based on the FGDs, *comfort‐smell* was understood as natural, familiar, just‐about‐right intensity, and harmony.
